# Genotypic difference in yield response to far-red light in dwarf tomato: explained by fruit sink and source strength and predicted by seedlings’ height plasticity

**DOI:** 10.3389/fpls.2026.1914198

**Published:** 2026-08-13

**Authors:** Maria Mastoraki, Leo F. M. Marcelis, Ep Heuvelink

**Affiliations:** Horticulture and Product Physiology, Department of Plant Sciences, Wageningen University and Research, Wageningen, Netherlands

**Keywords:** dwarf tomato, far-red, genotypic variation, *Solanum lycopersicum*, source–sink balance

## Abstract

Supplemental far-red (FR) light can enhance tomato yield and fruit soluble sugar content, yet its effects across genotypes remain poorly understood. The aims of this study were to determine whether genotypic differences in yield response to FR light are driven by dry matter production or partitioning and to identify an early predictive indicator (seed or seedling stage) of FR responsiveness in dwarf tomato genotypes. We studied 23 dwarf tomato genotypes for yield and fruit sugar responses to supplemental FR. To determine whether cultivars appearing non-responsive to FR exhibit stage-specific sensitivity, 2 non-responsive genotypes were exposed to FR during distinct developmental phases. In addition, seedling traits, including height, chlorophyll index, leaf morphology, and germination responses, were assessed to evaluate their potential to predict FR-induced yield responsiveness. We found that 10 of the 23 genotypes exhibited a significant increase in fruit fresh-weight yield under supplemental FR, ranging from 31% to 175% relative to the No FR treatment, whereas 13 genotypes showed no significant response. Similarly, 16 of the 23 genotypes exhibited a significant increase in total soluble sugar concentration, expressed on a dry-weight basis, under supplemental FR, ranging from 18% to 88% relative to No FR, whereas 7 genotypes showed no significant response. Enhanced yield responses were associated with increased fruit sink and source strength. Genotypes classified as non-responsive remained insensitive irrespective of the developmental stage of FR application, indicating true genotype-specific FR-insensitivity. Furthermore, seedling’s elongation response to FR was strongly correlated with the magnitude of yield enhancement under FR (r = 0.89). These findings demonstrate strong genotypic variation in tomato responses to supplemental FR and identify seedling height response as a promising early indicator of FR-induced yield responsiveness within the tested panel of dwarf tomato genotypes and controlled light conditions.

## Introduction

Light quality is a key environmental signal regulating plant development, morphology, and productivity. Among spectral components, far-red (FR) light, perceived primarily through phytochrome photoreceptors, has been extensively studied for its role in modulating shade-avoidance responses, photosynthetic performance, and reproductive development (Roig-Villanova and Martínez-García, 2016; Ballaré and Pierik, 2017; Zhen and Bugbee, 2020; Huber et al., 2021; Casal and Fankhauser, 2023; Huber et al., 2024; Li et al., 2011). Beyond its ecological relevance, FR supplementation has gained increasing interest in controlled-environment agriculture, where spectral optimization is used to enhance crop yield and fruit quality (Ji et al., 2020; Heuvelink et al., 2025).

Tomato (*Solanum lycopersicum L*.) is one of the most important horticultural crops worldwide and serves as a model species for studying light–plant interactions. Previous studies have demonstrated that FR supplementation can increase tomato yield and fruit soluble sugar content, largely through enhanced fruit sink strength (Kalaitzoglou et al., 2019; Kim et al., 2020; Ji et al., 2020; Vincenzi et al., 2025). However, these studies typically evaluated only one or two cultivars, limiting insight into potential genotypic variation in FR responsiveness.

Evidence for genotypic variation in FR responsiveness was provided by Ji et al. (2021), who evaluated 33 tomato genotypes and reported genotype-dependent growth responses to FR during the vegetative phase. Genotypes were classified as strongly, moderately, or weakly responsive based on changes in total plant dry mass. Importantly, this work was restricted to the vegetative stage and did not assess reproductive performance. Consequently, two key questions remain unresolved: (i) whether genotypic variability in FR responsiveness extends to the reproductive phase, thereby affecting yield and fruit quality, and (ii) whether we can predict from an early developmental phase which genotypes will have a significant increase in yield-related traits with the addition of FR and which genotypes will be less affected.

If genotypic differences persist during fruit development, an additional question arises regarding cultivars that appear neutral to FR. Specifically, it is unclear whether these cultivars are truly non-responsive or whether they exhibit stage-specific sensitivity that is mitigated when FR is applied continuously. We have previously shown (Mastoraki et al., 2025) that the developmental window from flowering to the full-grown green fruit stage is particularly sensitive to FR in terms of yield and sugar accumulation in fruits. However, it remains to be determined whether cultivars classified as non-responsive under continuous FR could respond when FR is restricted to a specific developmental window.

Thus, although FR responses in young tomato plants have been investigated across multiple genotypes, studies of fruiting plants have generally been restricted to a small number of cultivars. To our knowledge, the present study is the first to evaluate FR effects on fruit production, biomass allocation, fruit sink strength, and sugar accumulation across a broad panel of fruiting tomato cultivars and to test whether early developmental phase FR responses predict later yield responses.

The first aim of this study is to investigate if potential genotypic differences in yield response to FR are due to effects on production or partitioning of dry matter, and if potential effects on partitioning are due to effects on fruit sink strength and sugar metabolism. The second aim is to find an early predictive indicator (seed or seedling stage) of yield response to FR in dwarf tomato cultivars.

We screened 23 dwarf tomato cultivars to quantify genotypic variation in responses of yield and other phenotypic parameters to supplemental FR. Dwarf cultivars were selected because their compact architecture is suitable for limited growing space and vertical farming systems and enables replicated screening of many genotypes under controlled conditions. From this screening, we selected two cultivars that initially appeared neutral to FR and applied FR during distinct developmental stages to determine whether their apparent neutrality reflects true insensitivity or stage-dependent responsiveness. Finally, to evaluate the potential for early prediction of FR responsiveness, we quantified seedling traits- including seedlings height, chlorophyll index, leaf morphology, and germination responses- under FR and No FR conditions and assessed whether early trait plasticity correlates with later yield outcomes. Together, this work aims to clarify genotype-specific FR responses at the reproductive phase and to explore reliable indicators for selecting already in early plant developmental phase cultivars that show strong yield improvements resulting from additional FR.

## Materials and methods

### Plant material and general growth conditions

Twenty-three dwarf tomato (*Solanum lycopersicum*) cultivars (**Supplementary Table 1**) were grown in controlled-environment growth chambers at Wageningen University & Research (Wageningen, The Netherlands). Seeds were sown on stonewool plugs, and two weeks after germination, uniform seedlings were transplanted into 10 × 10 × 6.5 cm stonewool blocks (Grodan, Roermond, The Netherlands). Plants were distributed among independently controlled growth chamber compartments according to the experimental design described below.

Plants were grown under a 16 h photoperiod with a photosynthetic photon flux density (PPFD) of 200 μmol m^−2^ s^−1^ with a spectral composition of 24% blue (400–500 nm), 40% green (500–600 nm), and 36% red light (600–700 nm). In the supplemental FR treatment, 50 µmol m^−2^ s^−1^ of FR radiation (700–750 nm) was added to the same white-light background, resulting in a total photon flux of 250 µmol m^−2^ s^−1^ across 400–750 nm. Light intensity and spectral composition were measured once at the beginning of the experiment, 10 cm above the surface of the ebb-and-flow table. Light intensity was measured a second time at the top of the canopy of each plant when the fruits of the first truss had reached the red-ripe stage. Both measurements were performed using an LI-180 spectrometer (LI-COR Biosciences). Temperature and relative humidity were maintained at 22 ± 0.5 °C during the photoperiod and 19 ± 0.5 °C during the dark period, with 65% relative humidity.

Plants were cultivated on ebb-flood tables and all irrigated twice daily for 3 min with the same nutrient solution containing 1.2 mM NH_4_^+^, 7.2 mM K^+^, 4.0 mM Ca^2+^, 1.8 mM Mg^2+^, 12.4 mM NO_3_^−^, 3.3 mM SO_4_^2−^, 1.0 mM PO_4_^2−^, 35 μM Fe³^+^, 8.0 μM Mn^2+^, 5.0 μM Zn^2+^, 20 μM B, 0.5 μM Cu^2+^, and 0.5 μM MoO_4_^2−^. Solution electrical conductivity (EC) and pH were maintained at 2.1 dS m^−1^ and 5.5, respectively, and monitored twice a week. Flowers were manually pollinated three times a week using a Vibri Vario electronic bee (Royal Brinkman, ‘s-Gravenzande, The Netherlands).

### Experiment 1: Far-red induced change in fruit load, biomass allocation, and fruit carbohydrates

#### Light and fruit-load treatments

Twenty three dwarf tomato cultivars were grown under two light treatments: PAR light without supplemental FR (NO FR) and PAR light supplemented with 50 μmol m^−2^ s^−1^ FR (FR). Two fruit-load treatments were imposed. Under full fruit load, all fruits were retained to determine total fruit production and biomass allocation. Under the one-fruit-per-truss treatment, plants were pruned to retain three trusses, with one fruit retained on each truss. The resulting three fruits per plant were used to estimate potential fruit weight as an indicator of fruit sink strength (Marcelis, 1996).

#### Destructive measurements

To ensure that fruits used for destructive measurements were harvested at the same maturation stage across cultivars and treatments, fruit skin color was monitored twice a week using a portable colorimeter (CR-400, Konica Minolta, Tokyo, Japan). Fruits were harvested for analysis upon reaching cultivar-specific a*/b* threshold values corresponding to the ripe stage: 1.0 for red-fruited cultivars (red-ripe stage), 0.5 for orange-fruited cultivars (orange-ripe stage), and 0.11 for yellow-fruited cultivars (yellow-ripe stage). This approach allowed for standardization of physiological maturity, regardless of differences in cultivars and ripening time among treatments.

For plants subjected to the one-fruit-per-truss treatment, fruit fresh weight of each of the three retained fruits was measured at ripening. For plants maintained under full fruit load, all fruits were harvested at ripening and their fresh weights were recorded. Leaf area was quantified at the end of the cultivation cycle, following complete fruit harvest, using a LI-3100C leaf area meter (LI-COR Inc., Lincoln, NE, USA). Fruit, leaf, and stem dry weights were determined after drying samples in a ventilated oven for 24 h at 70 °C followed by 75 h at 105 °C.

#### Carbohydrate analysis

For carbohydrate analysis, the second and third fruits on the first truss of each full-fruit-load plant were harvested at the ripe stage and immediately sliced into two halves. One half was flash-frozen in liquid nitrogen and stored for biochemical analysis, whereas the other half was used for fresh and dry weight determination after drying in a ventilated oven (24 h at 70 °C followed by 75 h at 105 °C).

Frozen tissue of each individual sample was ground mechanically into fine powder with liquid nitrogen. Then, equal weights of powdered tissue from fruits harvested at positions 2 and 3 of the first truss of each plant were pooledand thoroughly mixed to generate one sample per plant, yielding in 3 independent mixed samples per light treatment. Glucose, fructose, and sucrose concentrations were measured as described by Plantenga et al. (2019) with an adaptation that 300 mg ground frozen fresh material from each pooled sample was weighed and mixed with 5 mL of 85% ethanol in a shaking water bath for 20 min at 80 °C. After centrifugation at 8500 g for 5 min, 1 mL of the supernatant containing soluble sugars was vacuum dried using a Savant SpeedVac rotary evaporator (SPD2010; Thermo Fisher Scientific, Waltham, MA, USA) and then dissolved in 1 mL Milli-Q water and diluted 50× for analysis of soluble sugars. Sucrose, fructose, and glucose quantification was conducted using a high-performance ion chromatograph (ICS-5000; Thermo Fisher Scientific) with an anion CarboPac 2 × 250 mm exchange column (PA1; Thermo Fisher Scientific) at 25 °C with 100 nM NaOH as eluent at a flow rate of 0.25 mL min^−1^. Pulsed amperometry was used for detection and Chromeleon (Thermo Fisher Scientific) was used for analysis of the chromatograms and quantification of sugar concentrations.

### Experiment 2: Developmental phase of far-red supplementation

#### Light treatments

Two dwarf tomato cultivars (*Heartbreaker Twiggy Red* (cultivar 11) from Prudac, the Netherlands, and *Balconi Red* (cultivar 22) from Graines-Voltz, France) were selected as non-responsive cultivars from Experiment 1 and grown under the general environmental conditions described above.

To investigate the effects of FR supplementation at different developmental stages, five FR treatments were applied, similar as in Mastoraki et al. (2025): No FR supplementation, FR supplementation throughout the entire growth cycle, FR from the vegetative stage until flowering, FR from flowering until full-grown green fruits, FR from the full-grown green fruits until fruit ripening.

In all FR treatments, supplemental FR intensity was 50 μmol m^−2^ s^−1^ and exposure duration was equal to 36 days. Thus, FR duration and intensity were maintained constant, while only the developmental timing of FR exposure varied among treatments.

#### Measurements

Total fruit fresh weight and fruit soluble sugar concentration were determined as described for Experiment 1.

### Experiment 3: Morphological responses to far-red light from seeds to young plants

#### Seedling Height

Seeds from the 23 dwarf tomato cultivars were germinated under the general growth conditions described above. Five days after sowing, seedlings were transferred to growth chambers providing either FR or NO FR light conditions. After two weeks of growth, seedlings’ height was measured using a digital caliper.

#### Leaf morphology and chlorophyll index

When plants reached the 6th true leaf stage, non-destructive measurements were performed on the 4th true leaf.

The number of leaf lobes was manually quantified on the 3rd and 4th leaflets of the 4th true leaf. Chlorophyll index was measured on the same leaflets using a Dualex sensor (FORCE-A, Orsay, France). Three chlorophyll measurements were taken per leaflet.

#### *In vitro* germination assay

Seeds of the 23 cultivars were surface-sterilized prior to *in vitro* germination by immersion in 1% (v/v) sodium hypochlorite solution supplemented with Tween-20 (three drops per 500 mL) for 20 min under intermittent agitation. Seeds were enclosed in sterile filter paper within a tea strainer during sterilization to facilitate handling and uniform exposure. Following sterilization, seeds were rinsed with sterile distilled water to remove residual disinfectant. Under sterile conditions, seeds were aseptically sown onto square petri dishes filled with germination medium consisting of Murashige and Skoog (MS) basal salts (2.2 g L^-1^), sucrose (10 g L^-1^), and Daishin agar (8 g L^-1^) without vitamins; the medium pH was adjusted to 5.8 using 1 M KOH prior to autoclaving.

Following stratification at 4 °C in darkness for 2 days, cultures were transferred to growth chambers under a 16 h photoperiod and either FR or NO FR light conditions as described above.

### Experimental design and statistical analysis

#### Experiment 1

In the first experimental run, 12 independent growth-chamber compartments were used. The four combinations of light treatment (FR or NO FR) and fruit-load treatment (full fruit load or one-fruit-per-truss) were randomly assigned to the compartments, with three replicate compartments per treatment combination. Each compartment contained one plant of each of the 23 cultivars, and plant positions were randomized within compartments. Thus, the compartment was the experimental unit for the effects of FR and fruit load, whereas the individual plants represented the different cultivars within each compartment.

The full-fruit-load treatment was repeated in a second independent run using 10 compartments, with five compartments assigned to FR and five to NO FR. The positions of the FR and NO FR treatments were changed between runs to avoid entangling of light treatment effects with compartment effects. Thus, total fruit production was evaluated using eight plants per cultivar and light treatment: three plants from the first run and five from the second run, with each plant grown in a different compartment. Total plant dry weight, harvest index, and soluble sugar concentration were measured only in the first run, using three plants per cultivar and light treatment.

The one-fruit-per-truss treatment was conducted only in the first run. Three plants per cultivar and light treatment were used, with each plant grown in a separate compartment. Each plant produced three measured fruits, resulting in nine fruits per cultivar and light treatment. The three fruit weights were averaged to obtain one plant level value; therefore, the statistical analysis was based on three independent plant values per cultivar and light treatment.

The full-fruit-load experiment was analyzed as a split-plot design using a linear mixed-effects model. Light treatment was applied at the compartment level as the whole-plot factor, while cultivar served as the split-plot factor, with one plant per cultivar grown in each compartment. An initial full model, Yield ~ Round × Treatment × Cultivar + (1 | Round: Compartment) was fitted to determine whether light treatment responses varied across experimental rounds. Because the round × light treatment and round × light treatment × cultivar interactions were not significant, they were removed from the model for the final analysis. Thus, the final analysis was based on the model: Yield ~ Treatment × Cultivar + (1 | Round: Compartment). Estimated marginal means, standard errors, and 95% confidence intervals were extracted using the *emmeans* package. To control the family-wise error rate across the 23 simultaneous cultivar assessments, P-values for the cultivar-specific contrasts were adjusted using the Holm method.

Variables measured in only one experimental run, including total plant dry weight, harvest index, soluble sugar concentration, and potential fruit weight under the one-fruit-per-truss treatment, were analysed using light treatment, cultivar, and their interaction as fixed effects. Compartment nested within light treatment was included as a random effect.

#### Experiment 2

The two selected cultivars were distributed across 10 independent growth-chamber compartments. Two compartments were assigned to each of the five FR treatments, and each compartment contained three plants per cultivar. Consequently, six plants per cultivar were evaluated under each FR treatment, distributed across two independent compartments.

Developmental FR treatment, cultivar, and their interaction were included as fixed effects. Compartment nested within FR treatment was included as a random effect and was considered the experimental unit for testing the treatment effect. Individual plants were treated as observations nested within compartments.

#### Experiment 3

The seedling-height experiment was conducted in two independent runs. In each run, two compartments were used, with one compartment assigned to FR and the other to NO FR. The treatment assignments were reversed between the first and second runs to balance possible compartment effects. Ten seedlings per cultivar and light treatment were measured in each run. These seedlings were considered subsamples and were averaged to obtain one value per cultivar, light treatment, and experimental run. Light treatment, cultivar, and the Light treatment × Cultivar interaction were included as fixed effects, with experimental run treated as a blocking factor.

The predictive performance of the relationship between seedlings plant-height response and yield response to supplemental FR was assessed using leave-one-cultivar-out cross-validation (LOOCV). In each of 23 iterations, one cultivar was excluded, a linear regression model relating yield response to plant-height response was fitted using the remaining 22 cultivars, and the fitted model was used to predict the yield response of the excluded cultivar. This procedure was repeated until each cultivar had been excluded and predicted once. The cross-validated predictive coefficient, Q^2^, was calculated as:


$${\text{Q}}^{2}=1-\left[\sum {\left({\text{y}}_{\text{i}}-{\hat{y}}_{\text{i}},\text{ LOOCV}\right)}^{2}/\sum {\left({\text{y}}_{\text{i}}-\bar{y}\right)}^{2}\right]$$

where y_i_ is the observed yield response of cultivar i, ${\hat{y}}_{\text{i}}$, LOOCV is the yield response predicted by the model fitted without cultivar i, and $\bar{y}$ is the mean observed yield response across all 23 cultivars (Westerhuis et al., 2008; Cruciani et al., 1992).

For the leaf-morphology and chlorophyll-index experiment, 10 independent compartments were used in one experimental run: five received FR and five received NO FR. Each compartment contained one plant of each cultivar, resulting in five plants per cultivar and light treatment. Light treatment, cultivar, and their interaction were included as fixed effects, whereas compartment nested within light treatment was included as a random effect. Measurements taken from multiple leaflets or multiple positions on the same plant were considered subsamples and averaged before analysis.

The germination assay was conducted in two independent runs. In each run, 11 seeds per cultivar and light treatment were sown in one Petri dish. Seeds within a dish were considered subsamples, and germination percentage was calculated at the Petri-dish level. Light treatment, cultivar, and the Light treatment × Cultivar interaction were included as fixed effects, with experimental run treated as a blocking factor.

For each seedling or germination trait, the cultivar level FR response was calculated as the percentage change relative to the corresponding NO FR value. Pearson correlations were used to assess associations between these cultivar level responses and the corresponding cultivar level fruit-yield response across the 23 cultivars.

Statistical analyses were performed in R version 4.3.1 using RStudio. Estimated marginal means and pairwise comparisons between light treatments within each cultivar were obtained using the *emmeans* package. Multiple comparisons were adjusted using the Tukey method. Statistical significance was assessed at P < 0.05. Figures were generated using the *ggplot2* package.

## Results

### Far-red-induced variation in yield is linked to fruit sink and source strength

**Figure 1:**
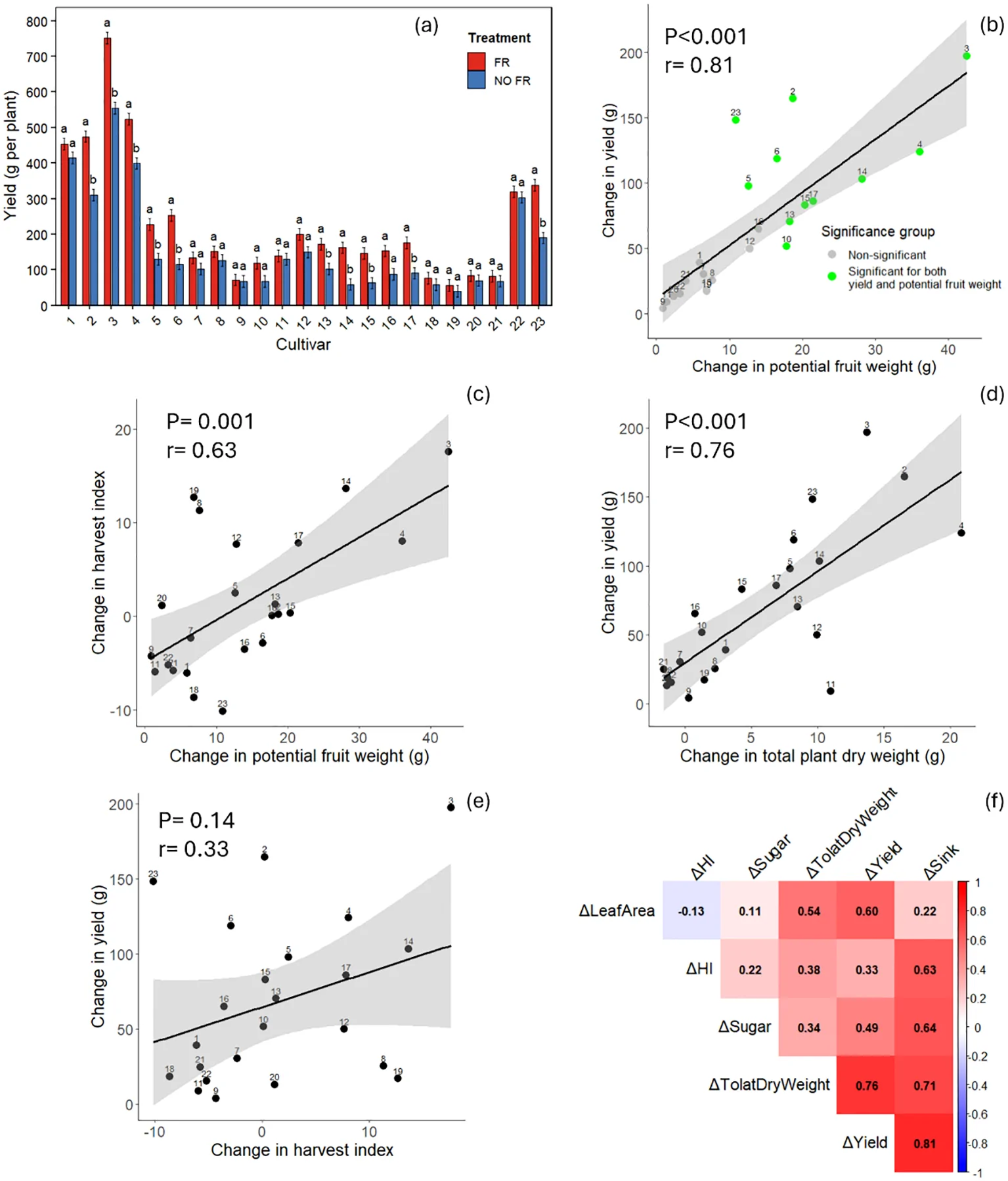
Effect of far-red (FR) light on yield, potential fruit weight, harvest index (HI), and total plant dry weight 23 dwarf tomato cultivars. **(a)** Yield (fresh weight of ripe fruits per plant) of plants grown under No FR (photosynthetically active radiation, PAR; 200 µmol m^−2^ s^−1^) or FR (PAR supplemented with 50 µmol m^−2^ s^−1^ FR). FR was applied from transplanting (four true-leaf stage) to harvest (red-ripe stage). Data represent estimated marginal means ± standard error (n = 8 plants per cultivar and light treatment). Different letters indicate significant differences between FR and NO FR within cultivars, based on cultivar-specific contrasts with Holm-adjusted P-values (α = 0.05). **(b, c)** Correlations between FR-induced changes in yield and HI and potential fruit weight across cultivars. **(d, e)** Correlations between FR-induced changes in yield and changes in total plant dry weight and HI, respectively. **(f)** Pearson correlation matrix of measured traits under FR and No FR conditions. Numbers indicate Pearson correlation coefficients (r), while color intensity reflects correlation strength; blue and red represent negative and positive correlations, respectively.

The effect of supplemental FR on fruit yield was evaluated in 23 dwarf tomato cultivars and was cultivar-dependent (**Figure 1a**). Ten cultivars exhibited a significant increase in yield (ranging from 31 up to 175%) under additional far-red (FR) light compared to No FR, whereas thirteen cultivars did not respond to FR supplementation.

This genotypic variation was closely associated with differences in response of fruit sink strength to FR, as assessed by potential fruit weight under a standardized one-fruit-per-truss condition. Cultivars that did not respond to FR in terms of yield also showed no response in potential fruit weight, whereas all responsive cultivars, apart from three, exhibited significant increases in both yield and potential fruit weight (**Figure 1b**). Accordingly, the magnitude of the FR-induced increase in yield was strongly correlated with the increase in potential fruit weight (r = 0.81; **Figure 1b**). Further analysis revealed that this variation in fruit sink strength was associated with variation in assimilate partitioning. Additionally, the FR-induced increase in harvest index (HI) was positively correlated with the increase in potential fruit weight (r = 0.63; **Figure 1c**), indicating that stronger responses of fruit sink strength to FR coincided with stronger responses of HI. Yield response was also strongly associated with change in total plant dry weight (r = 0.76; **Figure 1d**), while the comparatively weaker relationship between yield and HI (r = 0.33; **Figure 1e**) suggests a more limited contribution of partitioning alone. Together, these relationships indicate that genotypic differences in FR responsiveness were linked to variation in biomass production and fruit sink strength, rather than with one of these independently.

**Figure 2:**
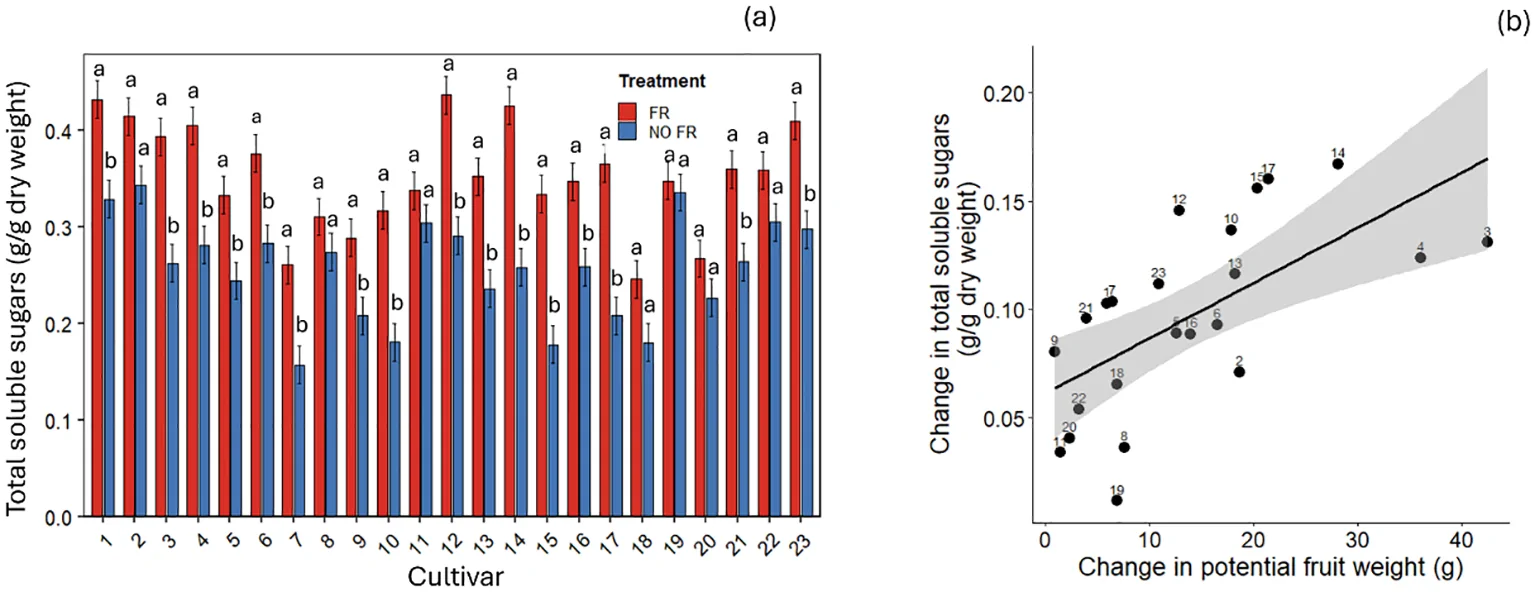
Total soluble sugar concentration in mature red fruits and correlation with the potential fruit weight of 23 dwarf tomato cultivars grown under two light conditions: No FR (photosynthetically active radiation, PAR; 200 µmol m^−2^ s^−1^) or FR (PAR supplemented with 50 µmol m^−2^ s^−1^ FR). Far-red light was applied from transplanting (four true leaves stage) until harvest (mature red fruit stage). **(a)** Data represent means ± standard error based on 6 fruits (3 independent mixed samples of 2 fruits each). Different letters indicate statistically significant differences between light treatments within cultivars according to Tukey’s HSD test (α = 0.05). **(b)** Correlation between the difference in fruit total soluble sugars and the potential fruit weight with and without FR.

To further characterize the physiological basis of these responses, fruit soluble sugar concentration was assessed under FR and No FR conditions (**Figure 2a**). The majority of cultivars (16 out of 23) exhibited a significant increase in soluble sugar concentration under FR (ranging from 18 up to 88%), while seven cultivars showed no significant response. Across cultivars, the FR-induced change in soluble sugar concentration was positively correlated with the change in potential fruit weight (r = 0.64; **Figure 2b**). This indicates that cultivars with a stronger increase in sink strength under FR also tended to accumulate higher levels of soluble sugars in the fruit.

### A tomato cultivar that does not respond to FR when applied throughout the whole cultivation period also does not respond to FR applied in specific developmental stages only

**Figure 3:**
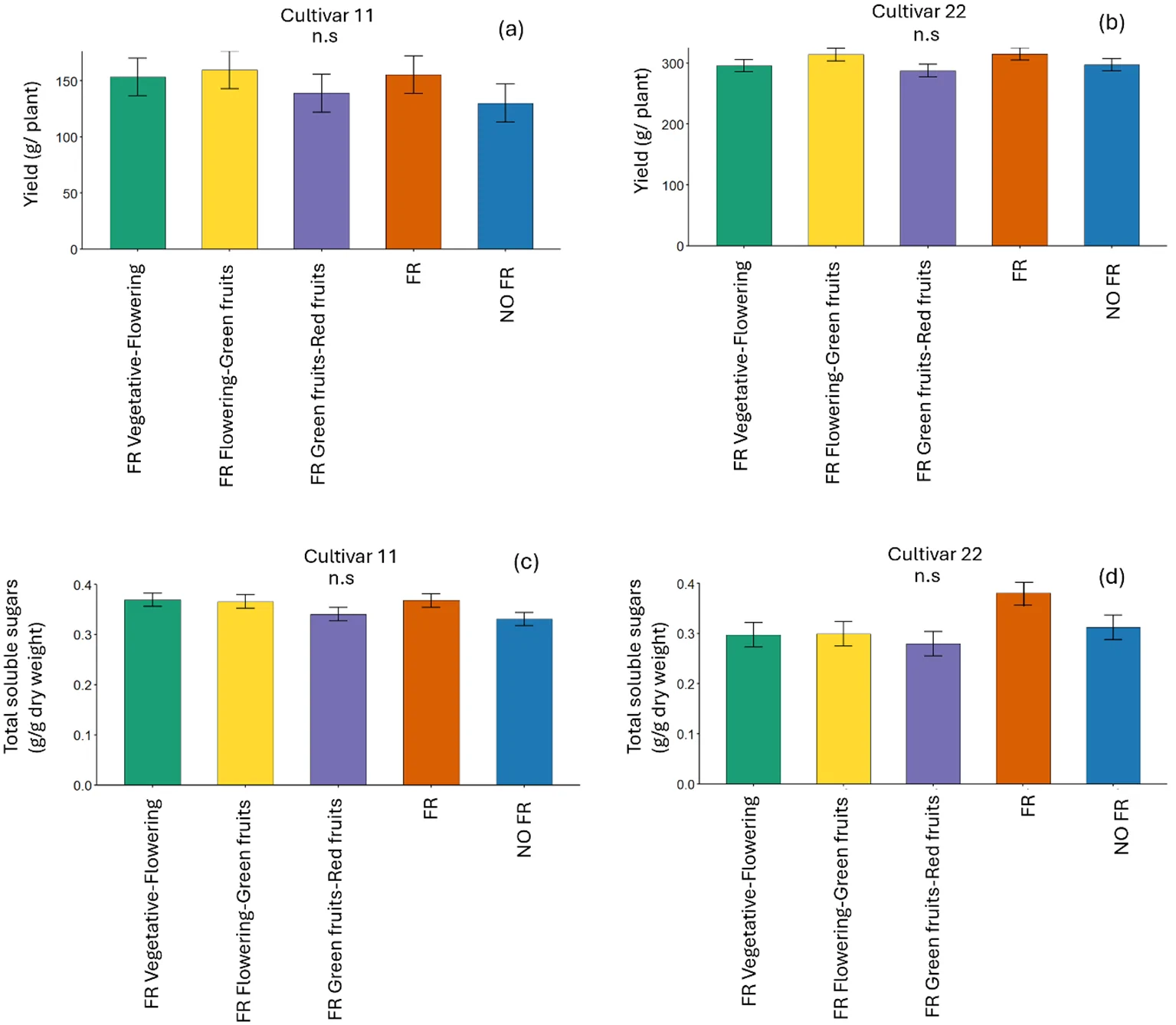
Effect of far-red light (FR) applied at distinct developmental stages on yield and total soluble sugars in dwarf tomato cultivars 11 and 22. These two cultivars were selected from the initial panel of 23 cultivars because they showed no significant response to continuous FR (from transplant to mature red fruit) in terms of yield and soluble sugars. FR treatments were therefore applied at specific developmental windows to further assess their responsiveness: vegetative to flowering, flowering to full-grown green fruit, green fruit to red fruit, throughout the entire growth period, or no FR. Panels **(a)** and **(b)** show yield per plant for cultivars 11 and 22, respectively, while panels **(c)** and **(d)** show total soluble sugar concentration in mature red fruits for cultivars 11 and 22, respectively. Data represent means ± standard error (n = 6). Different letters indicate statistically significant differences among treatments within each cultivar according to Tukey’s HSD test (α = 0.05). Effect sizes were estimated as the mean difference between each FR treatment and NO FR. For yield, estimated effects ranged from −1.0 to +16.2 g in cultivar 11, with Dunnett-adjusted 95% confidence intervals ranging from −51.3 to +66.6 g, and from −1.9 to +19.5 g in cultivar 22, with confidence intervals ranging from −41.7 to +59.3 (g) For soluble sugar content, estimated effects ranged from +0.0054 to +0.0311 in cultivar 11, with confidence intervals ranging from −0.0598 to +0.0963. In cultivar 22, stage-specific effects ranged from −0.0186 to +0.0131, whereas continuous FR produced the largest estimated increase (+0.0396; +12.8%; Dunnett-adjusted 95% CI: −0.0176 to +0.0968; P = 0.163).

To determine whether the cultivars that appeared unaffected by supplemental FR in terms of yield and sugar content were truly FR-neutral, or whether their response was influenced by the developmental stage that FR was applied, two cultivars classified as FR-non responsive for both traits were selected for further analysis (cultivar 11 and cultivar 22). In these cultivars, FR was applied at distinct developmental stages to assess stage-specific sensitivity. During none of the individual developmental stages, FR resulted in a significant increase in either yield or total soluble sugar content. Although a slight, non-significant trend toward higher yield and sugar content was observed when FR was applied throughout the entire growth period and from flowering to the full-grown green fruit stage, these differences were not statistically significant (**Figure 3**).

### Seedlings’ elongation plasticity is an early predictor of the magnitude of the effect of FR on tomato yield

**Figure 4:**
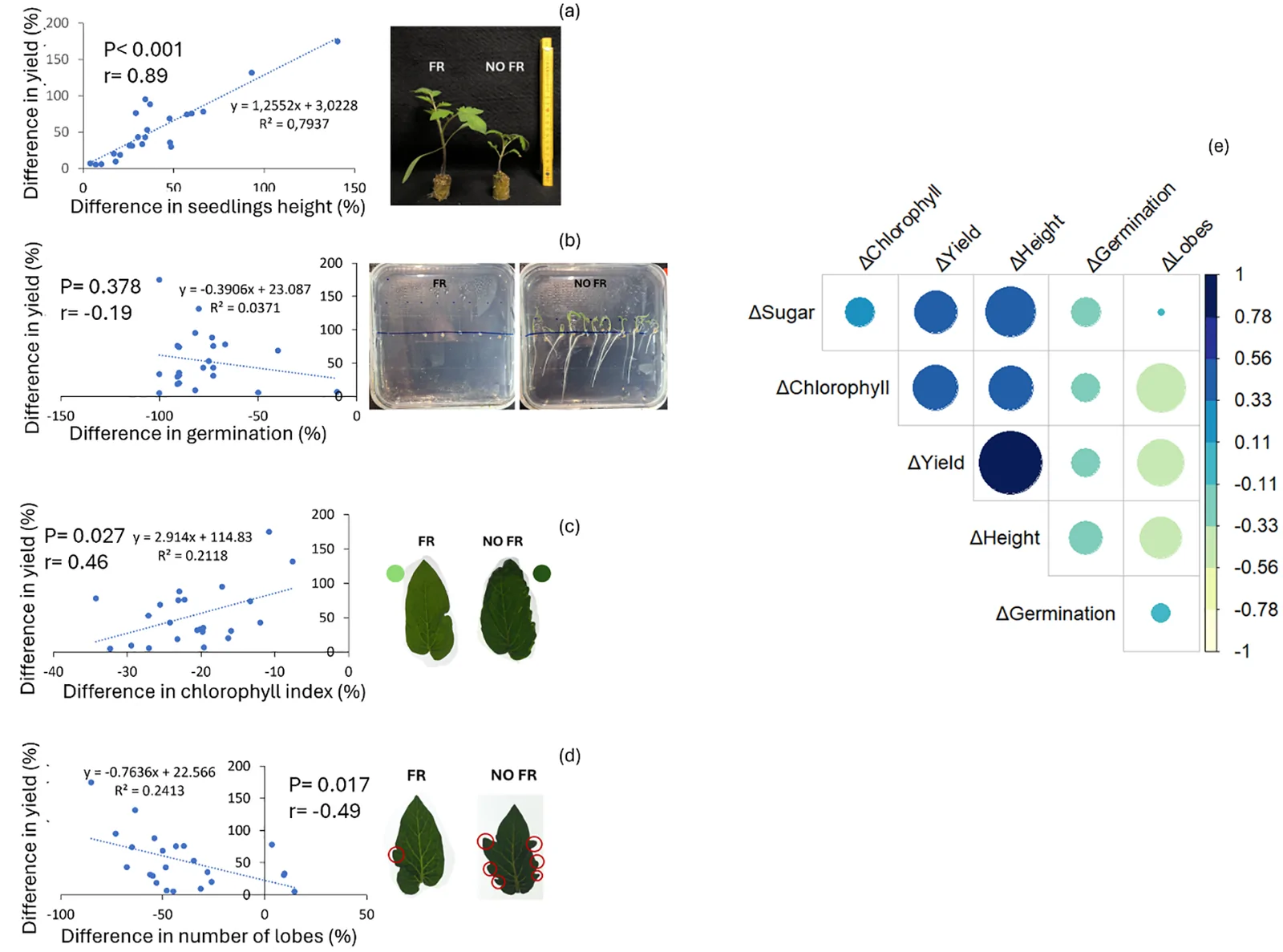
Correlation between early developmental traits and yield response under two light conditions: No FR (photosynthetically active radiation, PAR; 200 µmol m^−2^ s^−1^) or FR (PAR supplemented with 50 µmol m^−2^ s^−1^ FR) light in 23 dwarf tomato cultivars. Scatter plots show the relationships between **(a)** seedling height and yield, **(b)** germination rate and yield, **(c)** chlorophyll index (measured at the stage of six true leaves) and yield, **(d)** number of leaf lobes and yield. **(e)** collectively represents a correlation matrix showing the relationships between the four measured traits and yield. Circle size and color intensity reflect correlation strength, with larger circles indicating stronger correlations; yellow and blue denote negative and positive) correlations, respectively.

Having shown that the effect of supplemental FR on yield and sugar content is cultivar-dependent, and that cultivars classified as FR-unresponsive remain unresponsive regardless of the developmental stage at which FR was applied, we next examined whether early-stage traits could predict a cultivar’s responsiveness to FR in terms of yield. Among the measured traits, the difference in seedling height between FR and No FR showed a remarkably strong and highly significant correlation with the difference in yield between FR and No FR (r = 0.89, P < 0.001; **Figure 4a**). Yield response was also moderately positively correlated with the chlorophyll response (r = 0.46, P = 0.027; **Figure 4b**), but not with the germination response (r = −0.19, P = 0.378; **Figure 4c**). In contrast, the response in leaf lobe number was moderately negatively correlated with yield response (r = −0.49, P = 0.017; **Figure 4d**).

To further evaluate the predictive value of seedlings’ height responses to FR for yield responsiveness, cultivars were classified into three groups based on the percentage difference in seedlings’ height between treatments FR and No FR low (0-25% difference), medium (26-35%), high (36-150%). A similar classification was performed for the percentage difference in yield under the same conditions. The degree of correspondence between these classifications was then assessed to determine whether seedlings’ height response with and without FR could reliably predict yield response.

**Figure 5:**
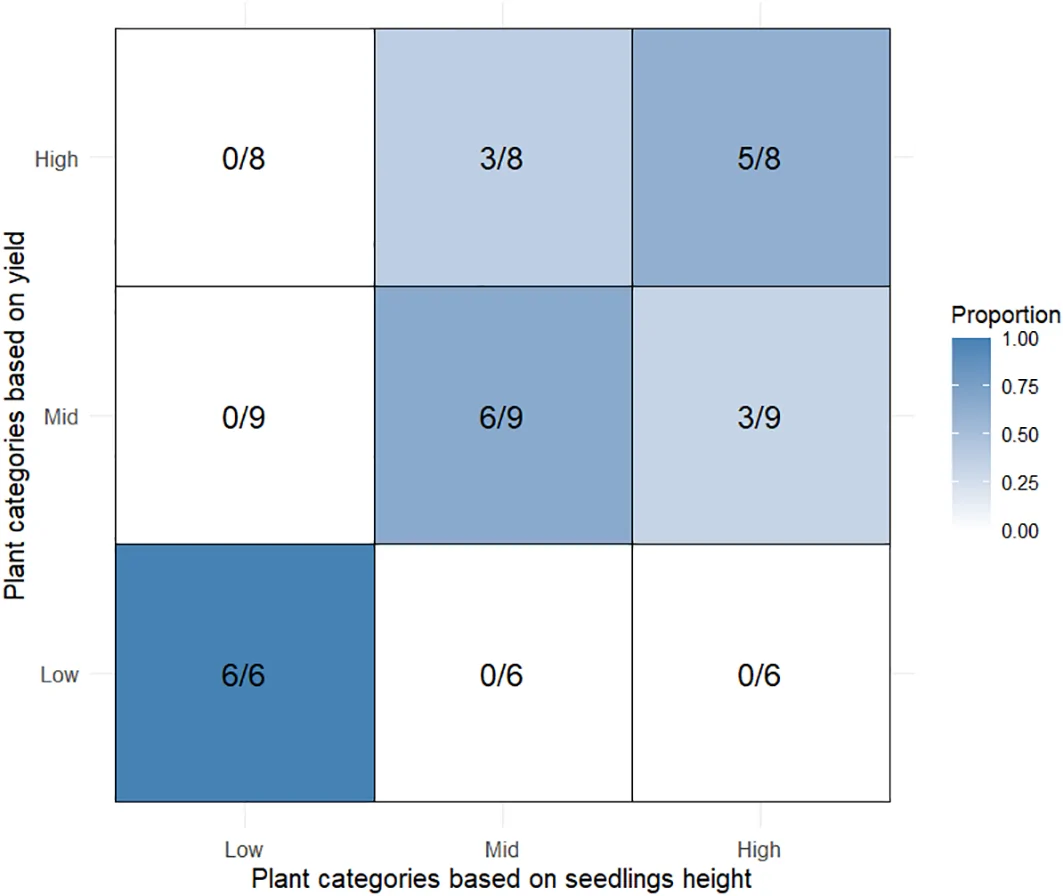
Association between seedling’s height categories and tomato yield categories across 23 cultivars. Tomato cultivars were grouped into low, medium (mid), and high categories based on the difference in seedling’s height measured with and without FR treatment and compared with corresponding difference in yield categories determined under the same treatments. The table shows the distribution of cultivars across yield categories (rows) as a function of seedling height categories (columns), based on differences between FR and No FR treatments.

Based on seedlings’ height differences, six cultivars were categorized as low, nine as medium, and eight as highly responsive to FR. Comparison with the yield-based classification revealed a strong accordance between the two traits (**Figure 5**). All six cultivars classified as low based on seedlings’ height differences were also classified as low in terms of yield response. Among the nine cultivars in the medium seedlings’ height group, six were also categorized as medium for yield, while three were classified as high. Similarly, of the eight cultivars assigned to the high seedlings’ height group, five corresponded to the high yield category and three to the medium category.

Overall, these results demonstrate a high level of agreement between seedlings’ height and yield based classifications, indicating that the seedlings’ height response to FR is a robust proxy for predicting cultivar-specific yield responsiveness. This approach therefore provides a practical and non-destructive tool for early selection.

## Discussion

### Far-red–induced yield responses are mediated by cultivar-dependent increases in fruit sink strength and biomass production

The present study demonstrates clear genotypic variation in the response of dwarf tomato cultivars to supplemental FR light (**Figures 1**, **2**). Whereas an earlier study (Ji et al., 2021), focused on the vegetative phase and identified variation among genotypes in traits such as plant height and early biomass accumulation, our work extends these findings to the reproductive stage. Specifically, we show that the effects of FR on yield and fruit soluble sugar content are strongly genotype-dependent. A subset of genotypes exhibited substantial increases in both yield (increases up to 175%) and sugar concentration (increases up to 88%) under FR, while in other cultivars these variables remained unaffected (**Figures 1a**, **2a**).

The genotypic differences in effects of FR light on yield observed in this study are strongly correlated with the genotypic differences in effects of FR on fruit sink strength (**Figure 1b**). This is consistent with the findings of Ji et al. (2020) for one cultivar, showing that FR increased yield as a result of increased fruit sink strength, and hence increased assimilate partitioning to the fruits. In the present experiment, this mechanism is extended to cultivar-dependent responses: cultivars that did not exhibit a significant increase in total yield under FR likewise showed no significant change in fruit sink strength, whereas cultivars showing a pronounced yield increase resulting from additional FR displayed also a substantial enhancement in fruit sink strength (**Figures 1b, f**). However, sink modulation alone does not fully explain the observed yield responses. Yield was also positively associated with total plant dry weight (**Figure 1d**), indicating that FR-driven increases in assimilate supply contribute to final productivity.

Because FR was added to the PAR light background rather than replacing part of it, plants in the supplemental FR treatment received a higher combined flux of PAR and FR photons than plants in the No FR treatment. Consequently, the observed responses cannot be attributed exclusively to FR-specific signaling and may partly reflect the greater total photon flux. Additional FR photons may affect photosynthesis and biomass production, while FR perception through phytochrome may regulate plant architecture, light interception and assimilate partitioning (Zhen and Bugbee, 2020; Jin et al., 2025). The observed yield responses may therefore result from a combination of increased photon supply and FR-specific signaling. Furthermore, differences in cultivar height and canopy proximity to the LEDs may have caused variation in the incident light intensity received by the plants. However, the relationship between the change in incident light intensity at the canopy surface and the corresponding yield response was weak (r = 0.32; R^2^ = 0.10; **Supplementary Figure 1**), suggesting that height-related differences in incident light explained only a limited proportion of the genotypic variation in yield response.

### A tomato cultivar that is not responsive to FR remains unresponsive regardless of the developmental stage that FR is applied

Consistent with the cultivar-dependent effects of FR on sink strength described above, 13 out of 23 cultivars showed no yield response, further highlighting strong genotypic variation in FR sensitivity. Based on previous findings identifying the period between flowering and the full-grown green fruit stage as a critical window for FR sensitivity in tomato (Mastoraki et al., 2025), we investigated whether restricting FR application to specific developmental stages could elicit responses in cultivars previously identified as non-responsive to FR. However, under the experimental conditions and with the available sample size, restricting FR application to particular developmental stages did not reveal clear evidence of a yield or soluble sugar response. In the two non-responsive cultivars tested, these findings suggest that cultivars unresponsive to FR under continuous supplementation remain largely unresponsive to FR-induced changes in yield and fruit sugar content, regardless of the developmental stage at which FR is applied. Thus, under the environmental conditions examined, FR responsiveness appeared to depend more strongly on cultivar identity than on the developmental timing of FR application.

### Seedlings’ elongation response to FR is an early predictor of the magnitude of the effect of FR on tomato yield

Numerous studies have developed models to predict tomato yield before harvest using morphological, phenological, and remote sensing traits. These approaches enable yield estimation at varying time points before harvest, with accuracy generally improving closer to harvest (Lillo-Saavedra et al., 2022; Tatsumi et al., 2021; Johansen et al., 2020; Rowland et al., 2020). In contrast, our study focused not only on predicting yield, but specifically on predicting cultivar responsiveness to FR light at a much earlier developmental stage.

Among the 23 cultivars evaluated, 10 exhibited a positive yield response to FR supplementation, whereas 13 showed no significant response (**Figure 1**). We identified a simple, early predictor, measurable just 2 weeks after germination, that distinguished between these response groups. Specifically, FR-induced seedling elongation was strongly associated with yield (**Figure 4a**). Although increased seedling height under FR is consistent with the well-established shade-avoidance response mediated by phytochrome B (phyB) inactivation (Franklin and Quail, 2010), we show that the magnitude of this elongation response is also strongly correlated with the magnitude of yield enhancement under FR (r = 0.89; **Figure 4a**). The robustness of this relationship was further supported by leave-one-cultivar-out cross-validation, which showed substantial predictive performance for yield response based on seedling height response (Q^2^ = 0.77; **Supplementary Figure 2**). Finally, all cultivars that showed no significant yield response to FR were assigned to the expected plant-height response category within this exploratory, in-sample classification (**Figure 5**). This result suggests that seedlings plant height responsiveness may help distinguish FR-responsive from non-responsive cultivars considering yield.

This correlation may reflect cultivar-specific differences in the perception or downstream transduction of FR signals. One possible explanation involves variation in the phytochrome B–phytochrome interacting factor (phyB–PIF) signaling pathway. Under low red to far-red conditions, inactivation of phyB can promote the accumulation and activity of Phytochrome Interacting Factors (PIFs), which regulate auxin and gibberellin related pathways associated with cell expansion and stem elongation (Lorrain et al., 2008; Li et al., 2016; Carriedo et al., 2016; Yang and Li, 2017; Li et al., 2024, Li et al., 2025). Cultivars showing a strong seedling elongation response may therefore also possess a greater capacity to respond to FR-mediated developmental signals later in the production cycle, potentially contributing to their stronger yield response.

Conversely, the limited elongation and yield responses observed in some cultivars could hypothetically result from lower sensitivity at one or more steps in FR perception or signal transduction, including phyB-mediated signaling, PIF activity, or downstream hormonal responses. This working hypothesis could be tested in future studies by comparing FR-responsive and non-responsive cultivars for phytochrome signaling activity, PIF expression and activity, and changes in auxin and gibberellin related pathways following FR exposure. Combining these measurements would help determine whether variation in the phyB-PIF-hormone signaling network contributes to the cultivar-dependent elongation and yield responses observed here.

Importantly, seedling height measurement is non-destructive and scalable to high-throughput phenotyping. Because it can be measured at a very early developmental stage it represents a promising candidate trait for identifying FR-yield-responsive genotypes before even fruiting has started. The difference in seeding’s height with and without FR represents a strong potential candidate indicator for not only distinguishing between responsive and unresponsive genotypes but also predicting the magnitude of the effect of FR on tomato yield. Finally, further studies using commercial tomato genotypes under greenhouse conditions will also be needed to determine whether the relationships identified here extend beyond dwarf tomato grown in controlled growth chambers. Such validation would provide the basis for its potential future application in breeding and screening programs.

## Conclusions

Within the context of dwarf tomato genotypes grown under controlled growth chamber conditions, our study demonstrates that: 1) genotypic variation in the responsiveness of both fruit sink and source strength to FR appears to be key factors underlying differences in yield stimulation. 2) A genotype that is unresponsive to FR in yield and fruit sugar content is unresponsive regardless of the developmental phase in which FR is applied. 3) The difference in seeding’s height with and without FR represents a strong potential candidate indicator for not only distinguishing between responsive and unresponsive genotypes but also predicting the magnitude of the effect of FR on tomato yield. Finally, further studies using commercial tomato genotypes under greenhouse conditions will be needed to determine whether the relationships identified here extend beyond dwarf tomato grown in controlled growth chambers.

## Data Availability

The raw data supporting the conclusions of this article will be made available by the authors, without undue reservation.
