# Supplementary material for: Genotypic difference in yield response to far-red light in dwarf tomato: explained by fruit sink and source strength and predicted by seedlings’ height plasticity
Source: Front Plant Sci. 2026 Aug 13;17:1914198. doi: 10.3389/fpls.2026.1914198 (PMC13519804; doi:10.3389/fpls.2026.1914198)
Supplement: Supplementary file 1 [file Supplementaryfile1.docx]

# Supplementary material

Table 1 Tomato cultivars used in this study, including cultivar name, breeding company, and fruit color.

| Cultivar number | Cultivar name | Breeding  company | Fruit color |  |  |
| --- | --- | --- | --- | --- | --- |
| 1 | Sweet Sturdy Jimmy | Prudac | Red |  |  |
| 2 | Sweet Sturdy Grace | Prudac | Red |  |  |
| 3 | Heartbreakers Vita | Prudac | Red |  |  |
| 4 | Pillar Catch Orange | Prudac | Orange |  |  |
| 5 | Pillar Tomatoes Joy Red | Prudac | Red |  |  |
| 6 | Pillar Tomatoes Joy Yellow | Prudac | Yellow |  |  |
| 7 | Ponchi Do | Prudac | Red |  |  |
| 8 | Ponchi Re | Prudac | Yellow |  |  |
| 9 | Ponchi Mi | Prudac | Red |  |  |
| 10 | Ponchi Fa | Prudac | Red |  |  |
| 11 | Heartbreaker Twiggy Red | Prudac | Red |  |  |
| 12 | Heartbreaker Twiggy Orange | Prudac | Orange |  |  |
| 13 | Ponchi Re Yellow | Prudac | Yellow |  |  |
| 14 | Plumberella Rosy-Pink | Graines-Voltz | Rosy-pink |  |  |
| 15 | Plumberella Red | Graines-Voltz | Red |  |  |
| 16 | Plumberella Gold-Orange | Graines-Voltz | Orange |  |  |
| 17 | Plumberella Creamy-Yellow | Graines-Voltz | Yellow |  |  |
| 18 | Funnyplums Red | Graines-Voltz | Red |  |  |
| 19 | Funnyplums Golden-Orange | Graines-Voltz | Orange |  |  |
| 20 | Funnyplums Rosy-Pink | Graines-Voltz | Rosy-pink |  |  |
| 21 | Funnyplums Creamy-Yellow | Graines-Voltz | Yellow |  |  |
| 22 | Balconi Red | Graines-Voltz | Red |  |  |
| 23 | Balconi Yellow | Graines-Voltz | Yellow |  |  |

Table 2 Cultivar-specific effects of supplemental far-red light on tomato yield. Values represent means ± SE for plants grown under FR and No FR conditions. Percentage change was calculated relative to the No FR treatment. Differences represent FR - No FR and are presented with 95% confidence intervals. P-values correspond to pairwise comparisons between light treatments within each cultivar. Significant differences were determined at P < 0.05.

Table 3 Cultivar-specific effects of supplemental far-red light on tomato fruit total soluble sugar content. Values represent means ± SE for plants grown under FR and No FR conditions. Percentage change was calculated relative to the No FR treatment. Differences represent FR - No FR and are presented with 95% confidence intervals. P-values correspond to pairwise comparisons between light treatments within each cultivar. Significant differences were determined at P < 0.05.

To determine whether the observed yield responses to FR were caused by effect of canopy height on incident light level, i.e. whether taller cultivars experienced stronger yield stimulation simply because they were physically closer to the LEDs, we quantified the relative change in incident light intensity at the top of the canopy and compared it with the relative change in yield for each cultivar (Figure S1). The correlation between the percentage increase in canopy light intensity and the percentage increase in yield was weak (r = 0.32), and the regression model explained only 10% of the variation in yield response (R² = 0.10). The resulting regression slope was shallow (y = 0.37x + 36.1), indicating that genotypes exposed to slightly higher light intensity due to their height did not consistently show a stronger yield increase. These findings suggest that the positive yield response to FR in this experiment cannot be attributed solely to differences in plant height or canopy proximity to the light source, supporting the conclusion that FR itself, rather than height-related variation in light exposure, contributed to the observed yield effects.

Figure S1 Relationship between the difference in light intensity measured at the top of the canopy and the yield response to supplemental far-red light across 23 dwarf tomato cultivars. Canopy level light intensity was measured for each plant when the fruits of the first truss had reached the red ripe stage. For each cultivar, the differences in canopy light intensity and yield were calculated between the supplemental FR and NO FR treatments. Each point represents one cultivar, and the dotted line indicates the fitted linear regression (R² = 0.10), showing a weak association between the difference in canopy light intensity and the yield response to supplemental FR.

Leave-one-cultivar-out cross-validation was used to evaluate the predictive robustness of the relationship between seedling height response and yield response to FR. For each cultivar, yield response was predicted from seedling height response using a linear model fitted to the remaining 22 cultivars. The model showed substantial predictive performance ($Q^{2}=0.77$), with an RMSE of 19.96 and an MAE of 14.03 percentage points, although some cultivars with intermediate to high yield responses were underpredicted (cultivars 6, 10, 17).

Figure S2 Leave-one-cultivar-out cross-validation of the relationship between seedling height response and yield response to supplemental far-red light across 23 dwarf tomato cultivars. For each cultivar, yield response was predicted from seedling height response using a linear regression fitted to the remaining 22 cultivars. The dashed line represents the 1:1 relationship between observed and predicted yield responses. Predictive performance was evaluated using the cross-validated Q^2^, root mean squared error (RMSE) and mean absolute error (MAE). Cultivar numbers correspond to those listed in Supplementary Table 1.
